# Does spatial variation in predation pressure modulate selection for aposematism?

**DOI:** 10.1002/ece3.3221

**Published:** 2017-08-15

**Authors:** S. Tharanga Aluthwattha, Rhett D. Harrison, Kithsiri B. Ranawana, Cheng Xu, Ren Lai, Jin Chen

**Affiliations:** ^1^ Key Laboratory of Tropical Forest Ecology Xishuangbanna Tropical Botanical Garden Chinese Academy of Sciences Mengla Yunnan China; ^2^ University of Chinese Academy of Sciences Beijing China; ^3^ World Agroforestry Centre, East & Southern Africa Region Woodlands, Lusaka Zambia; ^4^ Department of Zoology University of Peradeniya Peradeniya Sri Lanka; ^5^ Kunming Institute of Zoology Chinese Academy of Sciences Kunming Yunnan China

**Keywords:** conspicuousness, Danainae, fitness, mimicry, toxicity, warning signals

## Abstract

It is widely believed that aposematic signals should be conspicuous, but in nature, they vary from highly conspicuous to near cryptic. Current theory, including the honest signal or trade‐off hypotheses of the toxicity–conspicuousness relationship, cannot explain why adequately toxic species vary substantially in their conspicuousness. Through a study of similarly toxic Danainae (Nymphalidae) butterflies and their mimics that vary remarkably in their conspicuousness, we show that the benefits of conspicuousness vary along a gradient of predation pressure. Highly conspicuous butterflies experienced lower avian attack rates when background predation pressure was low, but attack rates increased rapidly as background predation pressure increased. Conversely, the least conspicuous butterflies experienced higher attack rates at low predation pressures, but at high predation pressures, they appeared to benefit from crypsis. Attack rates of intermediately conspicuous butterflies remained moderate and constant along the predation pressure gradient. Mimics had a similar pattern but higher attack rates than their models and mimics tended to imitate the signal of less attacked model species along the predation pressure gradient. Predation pressure modulated signal fitness provides a possible mechanism for the maintenance of variation in conspicuousness of aposematic signals, as well as the initial survival of conspicuous signals in cryptic populations in the process of aposematic signal evolution, and an alternative explanation for the evolutionary gain and loss of mimicry.

## 1 INTRODUCTION

Animal coloration is a classic example of the power of natural selection in action. Conspicuous aposematic (warning) signals are used by toxic animals to communicate their unprofitability to potential predators, while cryptic coloration (camouflage) is used by prey to conceal themselves from predators. It was widely believed that aposematic signals should be highly conspicuous and colorful, such as combinations of red or yellow with black (Arenas, Troscianko, & Stevens, 2014; Bezzerides, McGraw, Parker, & Husseini, 2007; Davis, Chi, Bradley, & Altizer, 2012). This is because such highly conspicuous colors convey a clear signal, as they are very different from concealed, cryptic colorations (Sherratt & Beatty, 2003) and stand out against heterogeneous environment with varying light conditions (Osorio & Vorobyev, 2005). However, contrary to these expectations, there is increasing evidence that aposematic signals are diverse and conspicuousness among aposematic species varies from highly conspicuous to near cryptic (Darst, Cummings, & Cannatella, 2006; Endler & Mappes, 2004; Merilaita & Ruxton, 2007).

Toxic species often converge on similar warning signals, which reduce predator learning costs and reinforce selection for innate signal recognition, while some nontoxic species mimic these warning signals to derive protection (Speed, 2001). Visually, similar aposematic Müllerian mimics and their nontoxic Batesian mimics in a given area form mimicry complexes or mimicry rings. Mimics can vary substantially in the degree to which they resemble their model (Kikuchi & Pfennig, 2013), and over large geographic areas, the same species may mimic a suite of different, locally available model species or mimics, and their models may not have perfectly overlapping distributions (Pfennig & Mullen, 2010). The fitness consequences of mimics with such different resemblance to their model in the wild populations are not known. Theoretically, the abundance of mimics should remain low compared to their models for model–mimic relationship to persist. Increasing mimic abundance has a negative effect on the fitness of the model, and therefore, the mimic fitness is thought to be density dependent (Pasteur, 1982; Rowland, Mappes, Ruxton, & Speed, 2010; Speed, 2001). However, limited data are available on model–mimic fitness in the field. Moreover, only a very limited understanding of how aposematic and mimetic signals evolve and spread over large geographic areas is available (Davis Rabosky et al., 2016). Both aposematism (Härlin & Härlin, 2003; Rudh, 2013; Wang & Shaffer, 2008) and mimicry (Davis Rabosky et al., 2016; Fiedler, 2010; Oliver & Prudic, 2010) are not end products but evolutionary dynamics, that include character transformation and predator–prey interactions, where aposematic and mimetic signals may be gained and lost multiple times within the same lineage. Reasons for the gain and loss of aposematic coloration are not well understood, but in poison dart frogs, loss of conspicuous coloration was found to be correlated with reduced body size (Rudh, 2013), while that of mimicry is often attributed to absence or local extinction of model species (Prudic & Oliver, 2008).

The relationship between toxicity and conspicuousness has received much attention, yet is still not well understood. As the cost of defense through accumulating toxin is usually high (Agrawal & Konno, 2009; Nishida, 1995), evolution should select the most effective advertisement (Mallet & Joron, 1999; Sherratt, 2008). In most cases, species have honest signals: Highly conspicuous species are relatively highly toxic (Davis et al., 2012) and toxicity positively correlates with conspicuousness (Cortesi & Cheney, 2010; Maan & Cummings, 2012; María Arenas, Walter, & Stevens, 2015). However, sometimes toxicity is even inversely correlated with conspicuousness (Wang, 2011). In Ladybird beetles, resource availability determines whether the correlation between conspicuousness and toxicity is positive or negative (Blount et al., 2012). In other situations, there appears to be a trade‐off between the costs of accumulating toxin and the costs of signaling. As a result, highly toxic species may be intermediately conspicuous, while moderately toxic species can be highly conspicuous to gain similar protection (Darst et al., 2006). Also, when the production costs of toxins are low and the costs of signaling are high, some toxic species may remain inconspicuous (Lindstedt, Huttunen, Kakko, & Mappes, 2011). Aposematic signals are believed to have evolved from cryptic ancestors that started to produce or accumulate toxin (Santos, Coloma, & Cannatella, 2003). However, the existence of closely related toxic species that vary substantially in their conspicuousness remains unexplained. For example, Danainae butterflies such as Monarch, *Danaus plexippus*, are well known for aposematism and their conspicuous warning signals. Nevertheless, African and Asian Danainae butterfly species vary tremendously in their color and conspicuousness (Su, Lim, & Kunte, 2015).

Predation pressure may be another important selection force for the conspicuousness of aposematism. Seasonal variation in predator communities has been shown to impose seasonally dependent selection pressure on aposematic signals of varying conspicuousness (Mappes, Kokko, Ojala, & Lindström, 2014). As aposematic and mimetic signals occur over large geographic areas (Davis Rabosky et al., 2016), spatial variation in the abundance and identity of predators could result in corresponding changes in predation pressure (Nokelainen, Valkonen, Lindstedt, & Mappes, 2014; Valkonen et al., 2012). Hence, we proposed the hypothesis that spatial variation in predation pressure drives conspicuousness‐dependent spatial variation in aposematic and mimetic signal fitness (Figure S1a, b).

Predators are known to show instinctive aversion to conspicuous colors and patterns (Ruxton, Speed, & Broom, 2009; Schuler & Hesse, 1985), where wariness and rejection are more likely with larger signals (Gamberale & Tullberg, 1998). Birds learn to reject highly conspicuous signals faster than less conspicuous signals (Lindström, Alatalo, Lyytinen, & Mappes, 2001; Lindstrom, Alatalo, Mappes, Riipi, & Vertainen, 1999; Yachi & Higashi, 1998), although there are arguments against this being accepted as a rule (Sherratt & Beatty, 2003). We predicted that attack rates on highly conspicuous species would be low compared to less conspicuous species when background predation pressures were low, as a result of innate avoidance of aposematic signals and quick learning by predators (Gamberale‐Stille & Guilford, 2003; Lindstedt, Lindström, & Mappes, 2009; Rowe & Guilford, 1996; Ruxton, Sherratt, & Speed, 2004; Schuler & Hesse, 1985). When familiar food is available, most insectivorous birds avoid conspicuous or unfamiliar food, even without learning its unprofitability (Marples, Roper, & Harper, 1998). However, some birds are known to try novel food items and sometimes attack toxic prey (McMahon, Conboy, O'Byrne‐White, Thomas, & Marples, 2014). The incidence of such behavior may increase when the competition for prey increases and pressure to avoid the possibility of losing a palatable prey item increases (McMahon & Marples, 2017; Ruxton et al., 2004). Therefore, we suggest the detectability of conspicuous signals may become disadvantageous when predators are more willing to attack (Bohlin, Tullberg, & Merilaita, 2008). Hence, we predicted a shift in relative success of signals from conspicuous to cryptic as predation pressure increases. Further, as a consequence of imperfect mimicry, we predicted that nontoxic mimics would experience higher attack rates than models and that mimic attack rates would increase more steeply with increasing predation pressure than model attack rates (Kikuchi & Pfennig, 2013) (Figure S1b). Last, we introduced another new hypothesis that, where several toxic models co‐exist, mimics should mimic the most successful (least attacked) model.

**Figure 1:**
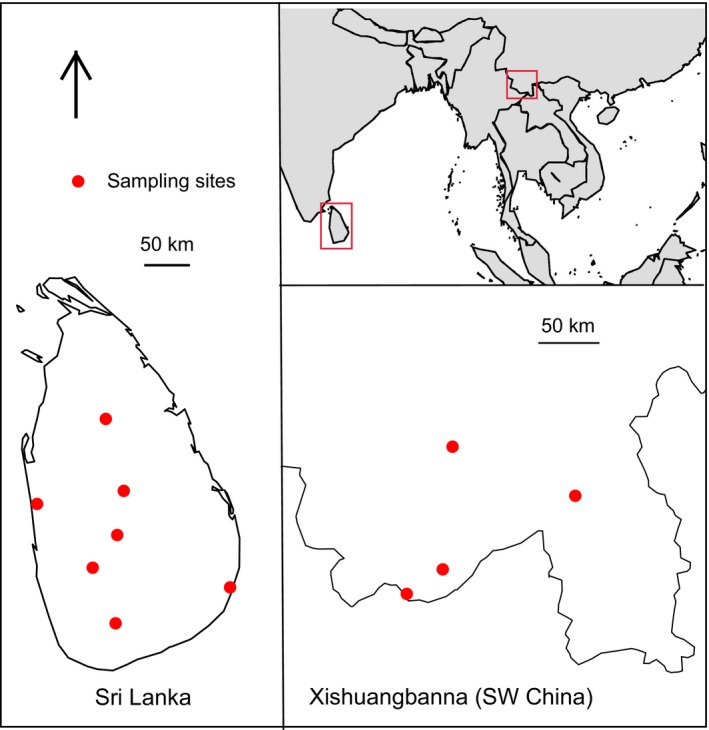
Distribution of sampling sites in Sri Lanka and SW China, Xishuangbanna. Sites were at least 50 km apart except for two sites in SW China, which were separated by deep valleys

**Figure 2:**
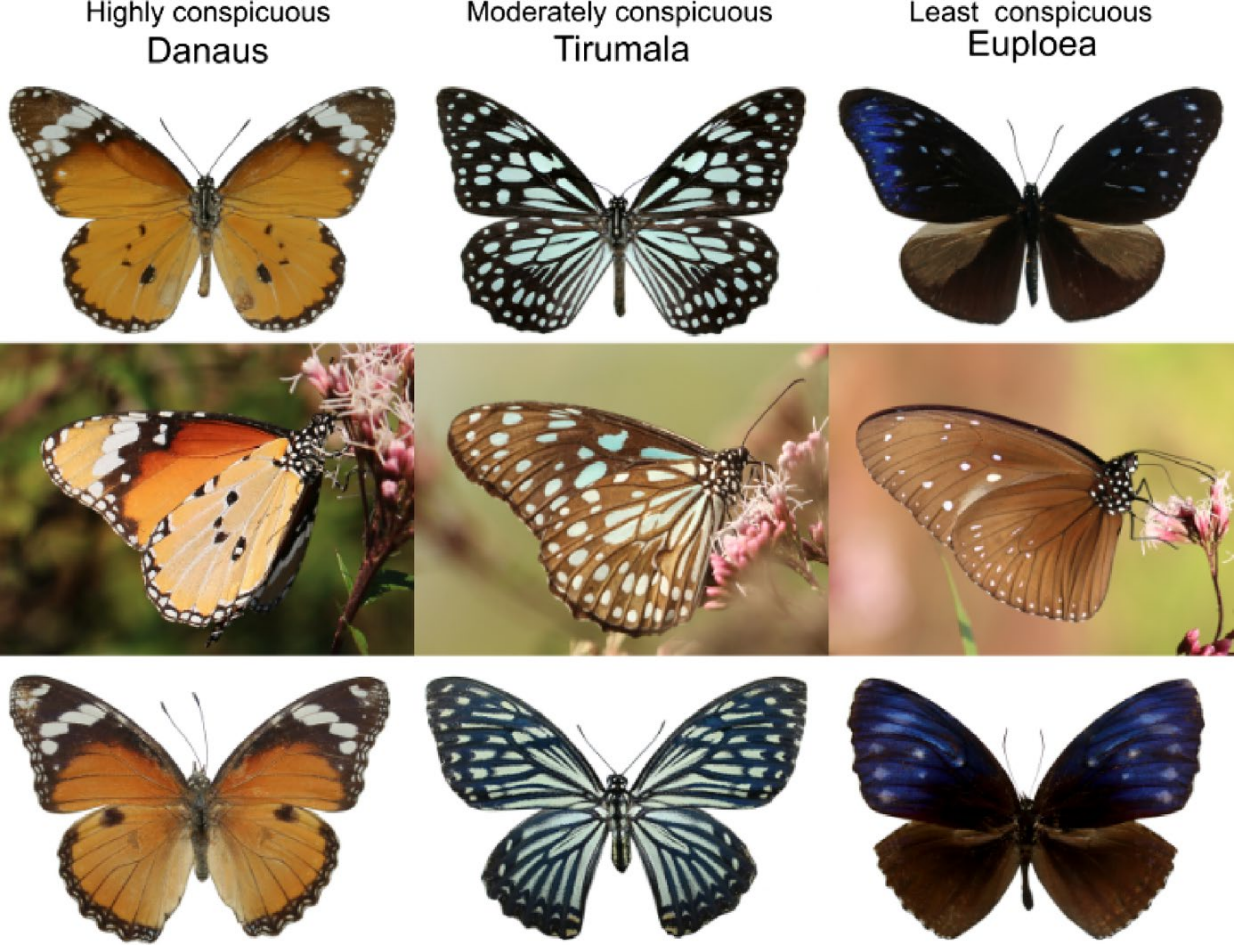
Example species from each of the three mimicry rings studied; (left to right) *Danaus genutia*,* Tirumala limniace*,* Euploea mulciber*. All three species were photographed in the same flower bed in SW China. First row: upper side of the wings. Second row: underside of the wings. Third row: mimic species of above model species; (left to right) *Hypolimnas misippus*,* Papilio clytia*,* Elymnias malelas*. Members of all three rings share the same habitats

To investigate these ideas, we selected three well‐known butterfly mimicry rings (*Danaus*,* Tirumala*, and *Euploea*; Nymphalidae, Danainae) mainly distributed across Asia from India, Sri Lanka to China and Japan. We investigated avian attack rates and abundance of selected species across 11 sites in Sri Lanka and SW China (Figure 1). Species in the selected Danainae mimicry rings vary substantially in their degree of conspicuousness. These butterflies can be visually separated based on differences in color, pattern, and overall conspicuousness (Figure 2), but share the same habitats, including roost and forage sites (STA's personal observations), are similar in size (with the wingspan of 70–100 mm) and have a similar characteristic slow flight. Moreover, danainae butterflies use chemical cues for intraspecific communication (Pliske & Eisner, 1969; Wyatt, 2003) and both male and female are visually identical. Therefore, the evolution of their conspicuousness can be reasonably attributed to aposematism, independent of sexual selection.

## 2 METHODS

### 2.1 Field survey

Our study system consisted of 11 sites in and around natural forests in Sri Lanka (seven sites) and SW China (four sites). SW China has an average temperature of 22 °C, mean annual rainfall is 1,500 mm, and the rain season is from May to October (http://www.ctfs.si.edu/site/Xishuangbanna). The dry zone Sri Lanka has 1,200–1,900 mm mean annual rainfall mainly from December to February, and the wet zone receives 2,500 mm during May–September. Mean annual temperature of sampling area in Sri Lanka varies from 26.5° to 28.5 °C (http://www.meteo.gov.lk/). Five transects, 250 m in length, were selected out of 8–10 areas of high butterfly activity within site covering an area of 25–100 km^2^ (Figure 1). Sites were at least about 50 km apart, and each site was visited four to six times at different periods of the year from 2012 to 2014 to control for any effect of seasonal variation, although such variations are minimal in the tropics. Each transect was sampled by two experienced observers (Polard walk, distance sampling) from 09.00 to 18.00, spending about 50 min per transect walk. Distances were marked on vegetation as an aid for estimation, and observers were pretrained for distance estimation. We aimed to sample each transect nine times, but due to inclement conditions, of 495 intended samplings a total of 451 samplings were completed. Observers and transects were shuffled randomly among sample days. All butterflies recorded within 30 m from the transect line were included. A total of 15,074 butterflies were surveyed. We also sampled birds along the same transect using 8 × 42 binoculars and identification of species were confirmed using three bird field guides (Harrison & Worfolk, 2011; MacKinnon, MacKinnon, Phillipps, & He, 2000; Robson, 2008). Birds were separated into their feeding guilds mainly based on handbook of the birds of the world (http://www.hbw.com/) supplemented by various other published literature.

### 2.2 Calculating attack rates and estimation of predation pressure

Butterfly species were separated into five categories: (1) aposematic models (i.e., toxic species, with aposematic coloration similar to at least one other co‐occurring model or mimic species); (2) mimics (i.e., nontoxic species that imitate the aposematic signal of co‐occurring models); (3) aposematic nonmodel (i.e., aposematic species with no mimic species imitating these butterflies); (4) toxic nonaposematic species (i.e., toxic species with no warning signals); and (5) nonmimic palatable species (i.e., nontoxic species without aposematic coloration). Species possibly belonging to mimicry rings were separated based on (Kunte, 2008; Su et al., 2015), and additional regional species were added based on visual similarity and host plant information. These included five mimicry rings (names based on the most common aposematic genus of the ring); *Danaus*,* Tirumala*,* Euploea* (Danainae: Nymphalidae), *Pachliopta* (Papilionidae), and *Delias* (Pieridae). Aposematic nonmodel species that are not mimicked by any other species (e.g., *Troides* spp.) and toxic species with no warning signals or for which the nature of aposematism is doubtful, such as *Ariadne merione* (Atluri, Bodapati, Rayalu Matala, Deepika Devara, & Reddi Chilakala, 2010), as well as Lycaenidae and some smaller Pieridae butterflies that probably have different predators (Ota, Yuma, Mitsuo, & Togo, 2014), were excluded from analyses. Other butterflies that feed on nontoxic plants were categorized as nontoxic, nonmimic species and were used to calculate the background predation pressure. Most of the aposematic species included in this study, at least all the genera, have been tested for toxicity in many previous studies. Apart from this available information, host plant information (including phytochemistry) of Asian butterflies collected by first author over 10 years reviewing literature, as well as by field and rearing observations, was used to support these groupings. When the host plant of a species was not known, information of closely related species or genus level information was used.

Predation pressure on aposematic species, such as poison frogs or snakes, has been tested using bite marks on artificial replica of these species (Valkonen et al., 2012; Willink, 2014). Similar methods have been applied to estimate predation pressure on resting lepidopteran, such as tiger moth where the species is immobile during daytime (Nokelainen et al., 2014). However, two‐thirds of attacks on butterflies occur when they are in flight and the remainder of the attacks occur when they are at rest where the wings are closed most of the time. Because of this, use of artificial replica to estimate predation pressure on butterflies is so far impossible. Therefore, we measured wing damage caused by bird attacks (beak marks and associated damage) on butterfly wings to estimate the predation pressure. Numerous studies have used the beak mark method (Ide, 2005; Kiritani, Yamashita, & Yamamura, 2013; Ohsaki, 1995; Ota et al., 2014; Shapiro, 1974) to assess predation pressure and arguments concerning the pros and cons of this method have been widely discussed in the literature (Kassarov, 1999; Ohsaki, 1995; Wourms & Wasserman, 1985).

Although when using this attack rate calculation killed or eaten individuals are not included, it has been shown that the proportion of butterflies that escape an attack can be used as a proxy for the predation pressure, especially for medium to large butterflies (Ide, 2005; Ohsaki, 2005; Ota et al., 2014). In this study, we compared the relative attack rate across the sites with respect to the background predation pressure, which was calculated as the attack rate on medium to large size (with the wingspan of >50 mm) nonaposematic, nontoxic butterfly species. Attacks on flying butterflies are distinguished as asymmetric damage (on one wing), while attacks on resting butterflies make symmetric damage (on both wings). Damage on a wing larger than 5 mm in depth is known to be caused by bird attacks. Damages of about 2–5 mm can be due to bird attacks or other natural causes. We categorized wing damage as very small (<2 mm), sharp‐edged, and smeared (damage across veins), which were also separated into symmetric or asymmetric. Symmetric wing damage was scored as a single attack. Very small damages were not included in beak mark damage count. The two indexes we developed, using the total number of damage marks on a specimen and scoring a specimen as damaged or not, were highly correlated (*p* < .000, *R* ^2^ = 93.3%). We categorized each specimen into an age class of five (1–5) and found ages were normally distributed. Therefore, whether the damage occurs at younger or older ages has no effect on the use of wing damage as an index. Furthermore, we used the binary index based on whether a specimen was attacked or not, because we considered that this was less likely to be affected by age. About 200 individuals of butterflies from each sampling site belonging to the different groups, as explained above, were collected and inspected for beak marks.

Birds are considered the main selective force for the evolution of aposematism in butterflies (Chai, 1986; Speed, Alderson, Hardman, & Ruxton, 2000; Svádová et al., 2009). Aposematism is often observed in medium to large butterflies, but there is no evidence that aposematism has evolved in small butterflies, such as Lycaenidae. Therefore, researchers often consider that the aposematic butterflies in a community have the same predatory birds (Chai, 1986). However, exact information on which bird species attack and consume which type or species of butterflies is very limited. Most of the empirical studies on aposematism have been limited to test animals such as Great tits, *Parus major*, or domestic chicken, *Gallus gallus*.

However, the insectivorous birds that are known to eat butterflies attack a wide range of butterfly species, including Danainae (Chai, 1986), and there is no evidence that the different aposematic species in the three mimicry rings under study have different predators. Predator density has been shown to correlate positively with the beak mark rate on the wings of medium (Nymphalidae and some Pieridae) and large (Papilionidae) butterflies (Ota et al., 2014). We found that both insectivore bird abundance, measured as encounter rate (*p* < .0001), and birds known to eat butterflies (*p* < .0001) were significantly correlated with attack rates in this study (Figure S3). However, we think attack rate is a better representation of predation pressure than the abundance of insectivorous birds.

Nontoxic, nonmimic butterfly species might have different predation prevention strategies such as flying fast or hiding in shadows. Such strategies may cause these butterflies to vary in the amount of predator attacks, but the lack of toxin or warning color as predator avoidance strategy and the larger sample size makes them suitable for calculating background predation pressure. In our survey, there was no evidence that composition of this butterfly group varied substantially among sites, although the presence of some rare species varied (See Table S4). Furthermore, we found that with increasing sampling size, the attack rate measured as proportion of attacks becomes very stable. Abundance measures, attack rates, and background predation pressure were calculated at the site level.

### 2.3 Quantification of warning signals

The message of a warning signal can be separated into conspicuousness and pattern complexity (Stevens & Ruxton, 2012). The distribution of conspicuous color on a cryptic background creates certain signal patterns. Symmetric patterns help species to stand out from a background, while asymmetric disruptive patterns help in camouflage (Forsman & Merilaita, 1999). Others have shown that movement and pattern can work in combination to create an aposematic signal (Rojas, Devillechabrolle, & Endler, 2014). Danainae butterflies have characteristic slow and uni‐directional flight which serves to educate predators (Chai, 1986) and a symmetric signal (Figure 2). The amount and distribution of dark color (black or brown) on the wing in combination with aposematic colors change the internal contrast of the signal (e.g., yellow color patch on black has higher contrast than yellow on brown). However, experiments have shown that predatory birds generalize the internal contrast and contrast against background and what is important is the overall color conspicuousness (Aronsson & Gamberale‐Stille, 2009). Therefore, we prepared a common tropical green foliage reflectance (by measuring the reflectance of leaves from sampling sites where most butterflies are found resting), as the background in the avian visual model to calculate conspicuousness, which was also weighted by the area of the color patch as the size of the signal affects avoidance learning (Forsman & Merilaita, 1999). We calculated the conspicuousness of all color patches (including darker black or brown patches) on butterfly wings against the green foliage background. When learning aposematic coloration, insectivorous birds first learn to avoid unprofitable color generalizing different patterns and shapes of the same color, and patterns and shapes are learned more slowly (Aronsson & Gamberale‐Stille, 2009; Kazemi, Gamberale‐Stille, Tullberg, & Leimar, 2014). Furthermore, the pattern similarity among members of the same mimicry ring has been found to be not as important as once thought (Rowe, Lindström, & Lyytinen, 2004).

Color pattern complexity was quantified from bitmap images using the Photoshop CS4 analysis tools and the statistical software R 3.2.3 (R Development Core Team, 2015). The area of warning color was considered one color, and the rest of the area was considered another color; therefore, in the pattern analysis, images had two colors (binarized image) (Miyazawa, Okamoto, & Kondo, 2010). The pattern simplicity score (PSS, inverse of complexity) is defined as the area‐weighted mean isoperimetric quotient of the contours (color patches) extracted from each image:

$$\text{PSS}=\sum_{i}w_{i}Q_{i}$$

where $Q_{i}=4\pi S_{i}/L_{i}^{2}$ is the isoperimetric quotient (or circularity) of each contour,

$$w_{i}=\frac{S_{i}}{\sum_{i}S_{i}}$$

*w*_*i*_ is the area weight; *S*_*i*_ and *L*_*i*_ are the area and the perimeter of each contour, respectively.

Butterfly specimens collected during our field sampling that were curated and deposited at Key Laboratory of Tropical Forest Ecology, Xishuangbanna Tropical Botanical Garden were used for reflectance measurements. Spectral reflectances were measured at 2 mm distance from six points of conspicuous regions of the butterfly wing using an Ocean Optics (Dunedin, FL, USA) PS2000 spectrometer, full‐spectrum light source (DT‐1000), Spectralon white standard, and reflectance probe (R400‐7). Conspicuousness of upper sides of wings of butterflies (as viewed from above by birds) was measured as the Euclidean distance (*E*) of color and brightness contrast, $E=\sqrt{\left(\Delta S^{2}+\Delta L^{2}\right)}$ where color = Δ*S* and brightness = Δ*L*, producing a vector distance in a perceptual space. A passerine tetrachromatic visual model (Vorobyev, Osorio, Bennett, Marshall, & Cuthill, 1998) that includes both chromatic (color) and achromatic (brightness) channel was used to evaluate the conspicuousness of the different color patterns. The avian vision model (Siddiqi, Cronin, Loew, Vorobyev, & Summers, 2004) was used to describe color (Δ*S*) and brightness (Δ*L*) discrimination, where vision is limited by photoreceptor noise.

The model starts by calculating the cone quantum catch (photoreceptor photon capture) *Q*_*i*_, for cone type (cone class), *i* is calculated as a function of the photoreceptor spectral sensitivity (*S*_*i*_), the irradiance spectrum incident on the color patch (*I*), and the reflectance spectrum of the patch (*R*) over the visible spectrum (i.e., 300–700 nm):

$$Q_{i}=\int_{\lambda =300}^{700}S_{i}\left(\lambda \right)I\left(\lambda \right)R\left(\lambda \right)d\lambda$$

This model assumes that photoreceptor adaptation follows the Weber–Fechner laws where, the receptor signal *f*_*i*_ of cone type *i* is proportional to the logarithm of the quantum catch;

$$f_{i}=\text{ln}Q_{i}$$

the difference in receptor signal *f*_*i*_ between two colors; for example, conspicuous wing color patch (A) and tropical green foliage (B) is therefore given by:

$$\Delta f_{i}=\text{ln}Q_{iA}-\text{ln}Q_{iB}=\text{ln}\frac{Q_{iA}}{Q_{iB}}$$

Noise in each receptor channel, ω_*i*_ known as Webber fraction, is derived from the noise‐to‐signal ratio of a single receptor, which is set by the relative number of receptor types within a typical avian receptive field (ω_*U*_ *= *1.0; ω_*S*_ *= *0.857; ω_*M*_ *= *0.520; ω_*L*_ *=* 0.515; where *U*, UV sensitive; *S*, short‐wave sensitive; *M*, mid‐wave sensitive and *L*, long‐wave sensitive) (Hart, Partridge, Cuthill, & Bennett, 2000).

The spectral distance ΔS in perceptual space is defined as,

$$\begin{array}{cc} {\left(\Delta S\right)}^{2} & ={\left(\omega_{U}\omega_{S}\right)}^{2}{\left(\Delta f_{L}-\Delta f_{M}\right)}^{2}\\ & +{\left(\omega_{U}\omega_{S}\right)}^{2}{\left(\Delta f_{L}-\Delta f_{S}\right)}^{2}+{\left(\omega_{U}\omega_{L}\right)}^{2}{\left(\Delta f_{M}-\Delta f_{S}\right)}^{2}+{\left(\omega_{S}\omega_{M}\right)}^{2}{\left(\Delta f_{L}-\Delta f_{U}\right)}^{2}\\ & +{\left(\omega_{S}\omega_{L}\right)}^{2}{\left(\Delta f_{M}-\Delta f_{U}\right)}^{2}+{\left(\omega_{M}\omega_{L}\right)}^{2}{\left(\Delta f_{S}-\Delta f_{U}\right)}^{2}/\\ & \left({\left(\omega_{U}\omega_{S}\omega_{M}\right)}^{2}+{\left(\omega_{U}\omega_{S}\omega_{L}\right)}^{2}+{\left(\omega_{U}\omega_{M}\omega_{L}\right)}^{2}+{\left(\omega_{S}\omega_{M}\omega_{L}\right)}^{2}\right) \end{array}$$

Brightness contrast (achromatic processing channel) of the avian visual system is calculated as a function of the double cone class that represents the absorption spectra of long‐wavelength sensitivity cone photoreceptors, *L* = *f*_*L*_ (Siddiqi et al., 2004). Brightness contrast estimates, ΔL, were evaluated as the absolute difference between two color elements:

$$\Delta L=(L_{1}-L_{2}/\omega_{L})$$

$$E=\sqrt{\Delta S^{2}+\Delta L^{2}}$$

Color and brightness values were calculated per specimen; when one species had two or more different color patches, for example, the orange and white patches in *Danaus* (see Figure 2) as well as the black or brown lines or margins, these values were calculated separately. Analysis of spectrum data and calculation of conspicuousness were conducted using the R package *pavo* (Maia, Eliason, Bitton, Doucet, & Shawkey, 2013).

### 2.4 Toxic analysis

Danainae butterflies are well known for accumulating toxic cardenolides from their larval host plants (Nishida, 1995, 2002). Cardenolides are cardiac toxins known to be toxic to both birds and mammals (Agrawal, Petschenka, Bingham, Weber, & Rasmann, 2012; Malcolm, 1991, 1994). These cardenolide compounds act as defensive chemicals in butterflies against their predators (Mebs, Zehner, & Schneider, 2000; Petschenka & Agrawal, 2015; Petschenka et al., 2013; Seiber, Lee, & Benson, 1983). Toxicity level has been used as a measure of noxiousness in aposematic studies combining warning signals and toxicity (Arenas et al., 2014; Blount, Speed, Ruxton, & Stephens, 2009; Darst et al., 2006). Birds use bitterness to estimate the level of toxicity (Skelhorn & Rowe, 2010), and cardenolides are bitter in taste (Malcolm, 1994). The level of toxicity correlates with the taste and birds learn to reject highly distasteful prey faster than less distasteful prey (Holen, 2013), and moderately toxic species are favored over highly toxic species in learning (Darst & Cummings, 2006). However, once learned, birds avoid prey types that are likely above certain level of toxicity or bitterness (Lindström, Lyytinen, Mappes, & Ojala, 2006). The drawback of the above studies lies in the use of relative toxicity measured among focus groups to understand the relationship between toxicity and conspicuousness or the relative difference in concentration of bitter substances, such as quinin. Together, these drawbacks make it difficult to compare results among studies. Mice as a common test animal have been used in aposematic toxicity analysis, while some other test species, such as water fleas (*Daphnia pulex*), are also used (María Arenas et al., 2015). Methods used to test toxicity also probably differ due to the level of toxicity and these methods often only give a quantitative measure of relative toxicity. Therefore, in this study, we decided to test for median lethal dose (hereafter LD50), which is the concentration of a substance needed to kill half of a population of test animals. LD50 is a common standard that can be used to compare different toxins, while calculating relative toxicity as well.

Therefore, the common model species of the mimicry rings studied (*Danaus genutia*,* Danaus chrysippus*,* Cethosia cyane, Euploea mulciber, Euploea core, Tirumala septentrionis, Tirumala limniace, Parentica aglea and Parentica melaneus*) were analyzed for toxicity. Crude toxins were extracted from wild‐caught butterflies from the field sites. For administration, crude toxins were extracted using a solvent (methanol) which was then evaporated off and the toxin was reconstituted with dimethyl sulfoxide (DMSO). KM mice were purchased from Kunming Medical University Laboratory Animal Centre. They were housed in a room with controlled temperature at 25 ± 2 °C and 50% ± 5% relative humidity with 12‐hr dark/light cycle. They were given free access to food and water ad libitum. The animals were acclimatized for at least 1 week prior to use. All the experiments using animals were carried out in accordance with recommendations in the Guide for the Care and Use of Laboratory Animals of Kunming Institute of Zoology, Chinese Academy of Sciences. All animal experiments described in this work were approved by the Institutional Animal Care and Use Committees at Kunming Institute of Zoology (2014‐204) (Permit Number: 33‐2397). Male KM mice between the weight ranges of 18–22 g were used in the acute toxicity study and intravenous (i.v.) administration of the toxins to mice was conducted. There was no previous absolute toxicity data of these toxins. Before determining the LD50 accurately, the proper dose range should be predicted. Therefore, three to four groups of five mice each were used to test the dose causing the death rate 0% and 100%, with 0.5 progression factor between adjacent groups. The appropriate dose range was chosen within the range of doses that were determined in the pretest for each toxin. Each toxin was diluted in menstruum (10 ml/kg saline solution containing 40% DMSO), employing an increasing dose with ratio of 1:0.85 between adjacent groups (e.g., 100 mg/kg, 85 mg/kg, 72.25 mg/kg etc.). These dilutions were i.v. administrated to 10 mice to obtain data to calculate LD50. The animals were kept under observation for 24 hr to register survival and death recorded every hour after the toxin was administered. The LD50 was determined according to improved Kou's method, which is designed to reduce the number of test animals needed (Tedford, Fletcher, & King, 2001). In total, the toxins from nine butterfly species were tested.

### 2.5 Data analysis

We used one‐way ANOVA to examine the effect of ring identity on conspicuousness, pattern simplicity score, toxicity, and wing hardness. To examine the factors affecting attack rates we used binomial generalized linear mixed‐effect models (GLMM) (Bolker et al., 2009). Independent variables included in the full model were background predation pressure, mimicry ring identity (=conspicuousness), model/mimic identity, relative abundance of mimics and nonmimic palatable species, and all two‐way interactions. Transect nested within site was included as a random variable. The abundance of aposematic and mimetic butterflies was also modeled using GLMM as a function of background predation pressure, ring identity, and model/mimic identity with transect nested within site as a random factor.

All statistical analyses were performed with the software R 3.2.3 (R Core Team, 2015) using the package *lme4* (Bates, Maechler, Bolker, & Walker, 2014). Graphs were generated using the packages *Effects* (Fox, 2003) and *Scidavis* (Benkert, Franke, Pozitron, & Standish, 2015). Maps were prepared using R packages *maps* and *mapdata* (Becker, Wilks, & Brownrigg, 2013). Effort was made to make the investigators blind to allocation during experiments and outcome assessment.

## 3 RESULTS

### 3.1 Conspicuousness and toxicity

**Figure 3:**
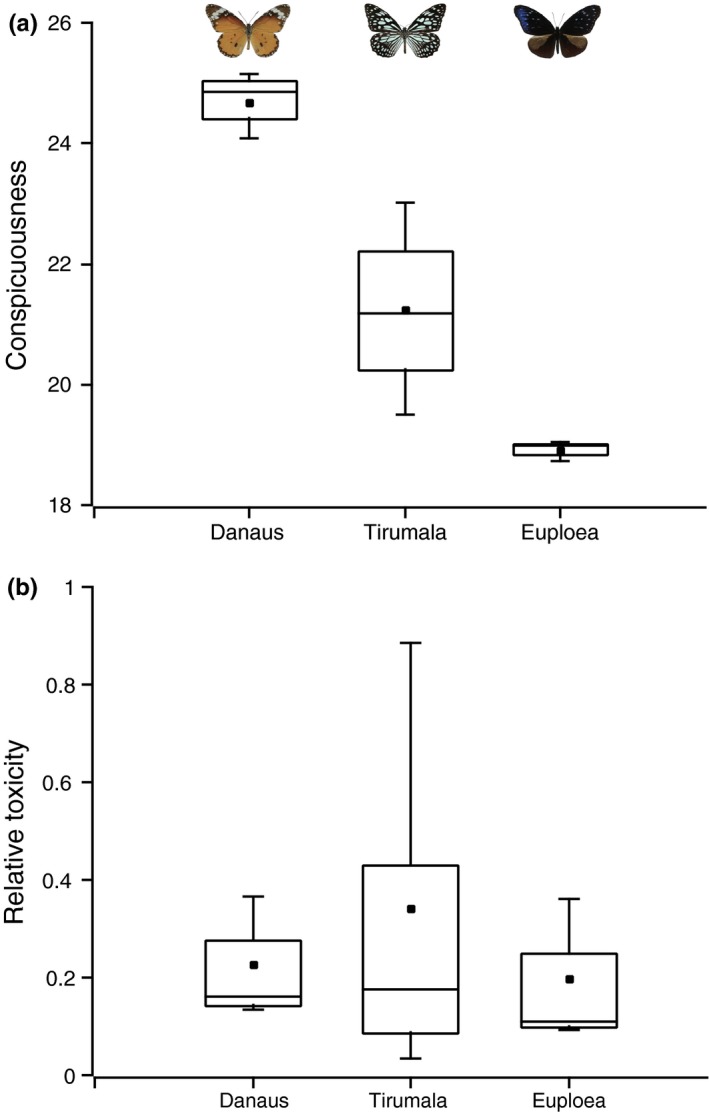
Characterization of aposematic signals. (a) Conspicuousness was evaluated as vector distance in perceptual space (Siddiqi et al., 2004) (*n* = 10). Nontoxic nonmimic species in the same habitats had conspicuousness values ranging from 23.98 to 18.77. (b) Quantification of toxicity using LD50 values; relative toxicity is presented with respect to the most toxic species (*n* = 9)

The three focal mimicry rings (*Danaus, Tirumala,* and *Euploea*) varied substantially in conspicuousness (Figures 2 and 3, Table S1). With respect to models, the relative area of conspicuous color on the wings and overall conspicuousness was highest in the *Danaus* ring and significantly decreased from *Tirumala* to *Euploea*. Pattern complexity also varied among butterflies within each of the rings, but variation among rings was not significant (*p* = .42, *F* = 0.93, Table S1). The conspicuous regions of the wings also comprised different sized fragments. *Danaus* had few relatively large fragments, *Tirumala* had many small fragments, and *Euploea* had a few very small fragments (Figure 2 and Figure S2). Small fragments are only visible at short range, while larger fragments can be seen from a distance. Area unweighted conspicuousness values of the small white fragments in *Euploea* were high (*Euploea* 58.48 ± 6.05 vs. *Danaus* 64.49 ± 21.48 and *Tirumala* 27.64 ± 6.02). So, although *Euploea* is inconspicuous at greater distances, these small fragments may have a strong short‐range warning effect, similar in strength to *Danaus*. Nevertheless, our results show that detection of the butterflies by the experienced observers involved in this study was not hampered by the variation in conspicuousness. Mean estimated distances of butterflies in the most conspicuous (Mean = 3.12, *SD* = 3.72) and the least conspicuous (Mean = 3.56, *SD* = 4.91) mimicry rings were not significantly different (*p* = .2033).

Toxicity varied within species (148.0 ± 88.8 mg/kg), but variation among models from the different rings was not significant (*p* = .64, *F* = .48; Figure 3b) (Table S1). Therefore, the models in all three rings may be described as adequately toxic and it is reasonable to conclude that the contribution of toxicity to avoidance learning by predators is similar.

### 3.2 Background predation pressure and attack rates

Across all 11 sites, we observed tremendous geographic variation in background predation pressure from 0.3 to 0.8 of individuals among nonaposematic, nontoxic species showing beak marks. Wing hardness of the study species was not significantly different among species (*p* = .5, *F* = .71, Table S1). Moreover, considering the bite force of insectivore bird beak (252–401 gf (Lederer, 1975)) and the hardness of butterfly wings (2.31–2.74 gf), it is unlikely that variation in butterfly wing hardness could affect bird beak mark rates. Among the examined individuals, 286 of 644 (502 models and 142 mimics) butterflies belong to three studied mimicry rings and 537 of 1,021 nontoxic nonaposematic butterflies had been attacked. All the species examined for wing damage were marked and released. Re‐captured individuals (very small number) were not included in the analyses. We examined how attack rates among the focal species (Table S4) varied spatially by fitting mixed effect models to the data that included the background predation pressure, mimicry ring identity, model or mimic identity, and abundance terms as independent variables. Transect nested within site was included as a random term in the model to account for the repeated measures design. We found that background predation pressure (95% CI = 5.143–13.832) contributed significantly to the variance in attack rates on butterflies belonging to the focal mimicry rings. Inclusion of the abundance of model or mimic species (95% CI = −0.560 to 8.988) or the relative abundance of palatable species (95% CI = −3.292 to 1.193) to the statistical model did not improve the explanatory power (Table S2 and Table S3a).

**Figure 4:**
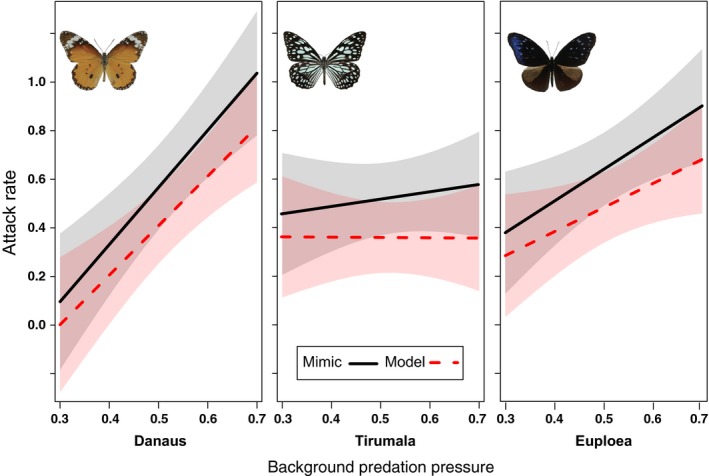
Effect of background predation pressure (PP) on attack rates of butterflies in three mimicry rings studied across 11 sites in Sri Lanka and SW China. Shaded area shows the 95% confidence intervals. Random effects such as sampling sites and fixed effects other than PP such as mimicry ring identity, model or mimic and their two‐way interactions were taken into account when PP vs. Attack rate effects were plotted

As predicted, butterflies in the three mimicry rings experienced substantially different patterns of variation in avian attack rates in response to changes in background predation pressure (Figure 4 and Figure S4). Models in the highly conspicuous mimicry ring (*Danaus*) experienced the lowest attack rates when background predation pressures were low, but the attack rate increased steeply with increasing background predation pressure, whereas models in the least conspicuous mimicry ring (*Euploea*) had a higher attack rate at low background predation pressures, but a less steep slope. Models in the intermediately conspicuous mimicry ring (*Tirumala*) experienced intermediate attack rates that did not change with increasing background predation pressure.

Also as predicted, the pattern was similar for mimics (Figure 2 and Figure S4), but attack rates on mimics were always higher than for models (95% CI = −1.398 to −0.102). Attack rates for mimics also increased more quickly with increasing background predation pressure than for models (Figure 3 and Figure S4b), but this result was not significant (95% CI = −1.177 to 0.051).

### 3.3 Background predation pressure and abundance of model and mimic butterflies

Among the 15,074 butterflies we recorded, 1,405 individuals of 16 aposematic model species and 61 individuals of 15 mimic species belonged to the three mimicry rings. Among these, five mimic species and six model species were present in all sites and were the most common species comprising 80% of all recorded individuals. Twelve species were rare and only recorded 1–5 times. Another six species were categorized as uncommon.

**Figure 5:**
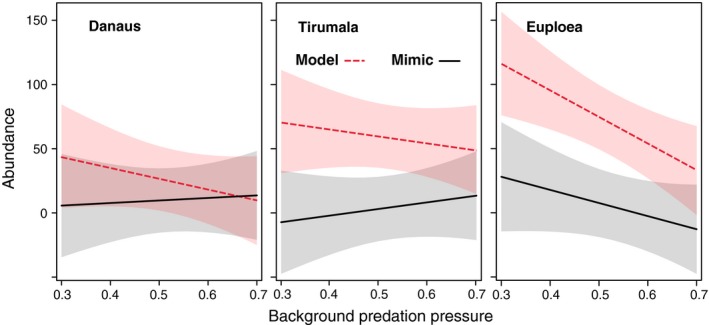
Variation of model and mimic species abundances along a background predation pressure gradient. Abundance of *Euploea* declined sharply. Mimic abundances were always lower than model abundances. Shaded area shows the 95% confidence intervals

We modeled the abundance of model and mimic butterflies as a function of background predation pressure, mimicry ring identity, model or mimic identity, and abundance terms. For all three mimicry rings, the abundance of models was always significantly higher than that of mimics (95% CI = 0.563–141.407; Figure 5). In addition, the abundance of *Euploea* models, which were the least conspicuous, declined sharply with increasing background predation pressure (95% CI = 6.645–94.001). The abundance of mimics in the other two mimicry rings also declined; however, the pattern was not significant. Background predation pressure did not have a significant effect on the overall abundance of the species in the three mimicry rings (95% CI = −107.721 to 147.234) and neither did ring identity (*Tirumala* 95% CI = −105.800 to 62.7118; *Euploea* 95% CI = −25.120 to 144.300) nor the interactive effect of ring identity and background predation pressure (*Tirumala* 95% CI = −114.813 to 178.952; *Euploea* 95% CI = −268.571 to 30.261). There was also no significant interactive effect of model/mimic identity and background predation pressure on abundance (95% CI = −227.659 to 13.302) (Table S3b).

In addition, our results illustrate some of the complex co‐evolutionary dynamics within mimicry rings. At the lower background predation pressure sites in our study, butterflies mimicked all three model types, whereas at the higher background predation pressure sites, species tended to mimic *Tirumala*, which had the lowest avian attack rates at these sites. For example, *Elymnias* females mimicked *Danaus* models in lower background predation pressure communities, but mimicked *Tirumala* at sites with higher background predation pressures, and *Elymnias* males were nonmimics in lower predation communities but mimicked *Euploea* when background predation pressures were high. Most strikingly, *Euploea* species also tended to mimic *Tirumala* at high background predation pressure sites. Females of *E. mulciber*, which occurred in high background predation pressure sites in SW China, have an aposematic signal that is intermediate between other *Euploea* and *Tirumala*. These qualitative observations deserve further attention.

## 4 DISCUSSION

In this study, three similarly toxic aposematic butterfly groups, that varied markedly in their degree of conspicuousness, experienced substantially different patterns of variation in avian attack rates in response to changes in background predation pressure. The study thus provides the first empirical evidence for a possible mechanism that can explain the maintenance of aposematic signal diversity independent of variation in toxicity. Background predation pressure‐dependent spatial variation in aposematic signal fitness can maintain aposematic signal diversity. Our study provides the first field evidence that mimics suffer higher attacks, causing the theoretically expected lower abundance, as compared to their models. Most strikingly, nontoxic Batesian mimics as well as some toxic Müllerian co‐mimics tended to imitate the warning signal of the least attacked model with higher fitness at any particular site.

As predicted, the most conspicuous butterflies in the *Danaus* ring with larger warning signal patches experienced very low or zero attack rates at sites with low background predation pressure in our study, presumably because the *Danaus* ring benefits from both innate avoidance and aversion learning by predator. However, the detectability of conspicuous signals became disadvantageous when predators started to attack unselectively, leading to a sharp increment of attack rates on the *Danaus* ring with increasing background predation pressure. In contrast, the least conspicuous, unpalatable species in the *Euploea* mimicry ring gained an advantage from crypsis at high background predation pressures, but benefitted less from innate predator avoidance at low background predation pressures. The protection of *Tirumala* apparently arises from the balance of moderate innate avoidance and avoidance learning by predators, and moderate camouflage, which was maintained across the predation pressure gradient. Theoretical modeling also indicates that beyond a certain level further increments in conspicuousness does not increase the protection, because of the trade‐off with increasing detection rates (Ruxton et al., 2009).

There are many factors affecting the abundance of butterfly species including aposematic and mimetic species. If attacked and caught, birds are known to release toxic prey with limited damage after learning its unprofitability (Skelhorn & Rowe, 2006). Similar observations have been made in experimental tests (Halpin, Skelhorn, & Rowe, 2008). When birds were presented with artificial toxic prey, both red (conspicuous) and brown (cryptic) prey were attacked, whereas the majority of red colored prey were released, the majority of brown colored prey were eaten (Halpin et al., 2008). The same study demonstrated that birds are unable to learn to avoid brown colored prey despite their toxicity. Such a process may have contributed to the declining abundance of butterflies in the *Euploea* ring with increasing background predation pressure. Other factors such as predation on caterpillars or pupae and availability of host plants are also likely to affect the abundances of butterflies, which may have caused the patterns of abundance in *Danaus* and *Tirumala* not to be significantly related to background predation pressure.

As hypothesized, mimics experienced higher attack rates than their models, which is most likely explained by behavioral and morphological differences resulting in imperfect mimicry. Although mimic species have developed an accurate or loose imitation of aposematic signals, other aspects of morphology may constrain close mimicry to their model species. Moreover, when mimicry is limited to a sex or a morph, their behavior and niche including food sources might differ from their model species. For example, *Elymnias* (Palmfly butterflies) species show mostly female‐limited mimicry and both sexes feed mainly on overripe fruits, while their model species are nectar feeders (Woodhouse, 1942). In addition, the relative abundance of mimics was always about 10% or lower that of the models. Contrary to our predictions, we did not detect a significant interactive effect of model/mimic identity and background predation pressure on either attack rates or abundance. However, the low abundance of mimics reduced the statistical power of our tests.

Recent advances in animal vision models have produced a better understanding of aposematic coloration (Brunton & Majerus, 1995; Chiao, Vorobyev, Cronin, & Osorio, 2000; Delhey, Delhey, Kempenaers, & Peters, 2014; Endler & Mielke, 2005; Maia et al., 2013; Osorio & Vorobyev, 2005; Vorobyev et al., 1998). However, as mentioned previously comparison of toxicities and the inferences drawn from different studies is hampered by the different methods that have been used to estimate toxicity levels, as well as the differences in units or measures. In many experiments, the different levels of toxicity have been estimated or are relative measures specific to particular studies. Therefore, we cannot predict how much toxin is needed for aversion learning. Future studies should focus on understanding natural predators of aposematic and mimetic species, developing better predation pressure estimation methods and measuring comparable toxicity values.

Field studies on spatial variation in aposematic signals have shown that variation in the patterns of warning signals can exist in adjacent populations, but the spatially restricted predators immediately learn the unprofitability of the artificially introduced exotic morph from other populations (Chouteau and Angers, 2011). The current study revealed a tremendous variation in predation pressure (30%–80% attacked) across a large geographic area in South and East Asia, which is also supported by global patterns of predation gradients (Roslin et al., 2017). Local variations in predator community composition and differences in predation pressure have also been shown to be an important driver of diversity in aposematic signals (Mochida, 2011; Nokelainen et al., 2014; Valkonen et al., 2012). Theoretical modeling (Endler & Mappes, 2004) and field experiments with artificial prey (Willink, 2014) have shown that a predator's willingness to attack different colors or conspicuousness may vary leading to variations in warning signal. These findings comply with our results and the absence of a Batesian mimicry complex for relatively less toxic, less conspicuous Müllerian mimicry systems, such as *Pieris* sp (family Pieridae), suggest that mimic species favor imitating models of higher fitness (Nishida, 2002; Rothschild, 1979). We observed that species tend to mimic the model of higher fitness. Such evidence suggests how shifts of aposematic model, as well as transitions between crypsis and mimicry, may occur. A novel hypothesis was introduced in this study, based on previous findings concerning predator behavior, learning, and variation in predation pressure and the hypothesis was tested with field data. Our quantitative field study further confirms most predictions of classical mimicry theory and laboratory experiments on aposematism, demonstrating the reliability of our study system.

However, field experiments have limitations in terms of standardization and involve assumptions. Therefore, future studies should focus on designing laboratory experiments, mathematical models, and computer simulations to test the predator behavior and fate of various aposematic signals along a predation pressure gradient. Furthermore, evolutionary rates and genetic variation along such gradients will be important to understand.

Since the introduction of mimicry theory by Bates (1862) with his pioneering work, aposematism and mimicry studies on Amazonian butterflies have contributed substantially to the understanding of natural selection and organic evolution. Wallace (1866) introduced the idea of warning signaling (later termed aposematism) during his studies of butterflies in tropical Asia, but further use of tropical Asian butterflies for aposematism and mimicry studies has been extremely limited (Ex: Yamauchi, 1993; Kitamura & Imafuku, 2010; Kunte et al., 2014; Su et al., 2015).

Furthermore, this study has provided insights into the understanding of how the fitness of different aposematic signals can change along a predation pressure gradient. Our hypothesis provides a mechanism for maintaining the diversity of aposematic signals, as well as supporting the initial survival and gradual evolution of conspicuous aposematic signals, and enabling evolutionary transitions between cryptic and conspicuous mimetic signals.

## AUTHOR CONTRIBUTIONS

S.T.A., J.C., and R.D.H. designed the experiment; S.T.A. and K.B.R. performed the field sampling; S.T.A., C.X., and R.L. conducted toxicity test; S.T.A. conducted data analysis; S.T.A., J. C., and R. D. H. wrote the manuscript; all authors commented on the manuscript.

## CONFLICT OF INTEREST

None declared.
