# Supplementary material for: Does spatial variation in predation pressure modulate selection for aposematism?
Source: Ecol Evol. 2017 Aug 15;7(18):7560–72. doi: 10.1002/ece3.3221 (PMC5606884; doi:10.1002/ece3.3221)
Supplement: Supplementary file 1 [file ECE3-7-7560-s001.docx]

**Supporting information**

**Does spatial variation in predation pressure modulates selection for aposematism?**

S. Tharanga Aluthwattha^1,2,‡^, Rhett D. Harrison^3^, Kithsiri B. Ranawana^4^, Cheng Xu^5^, Ren Lai^5^, Jin Chen^1,^*

^1^Key Laboratory of Tropical Forest Ecology, Xishuangbanna Tropical Botanical Garden, Chinese Academy of Sciences, Mengla, Yunnan 666303, China; ^2^University of Chinese Academy of Sciences, No. 19A Yuquan Road, Beijing 100049, China; ^3^World Agroforestry Centre, East & Southern Africa Region, 13 Elm Road, Woodlands, Lusaka, Zambia; ^4^Department of Zoology, University of Peradeniya, Sri Lanka; ^5^Kunming Institute of Zoology, Chinese Academy of Sciences, 32 Jiaochang Donglu, Kunming, Yunnan 650223, China

^‡^ aluthwattha@yahoo.com

^*^chenjin@xtbg.ac.cn

**Contents**

**Figure S1.** Explaining signal diversity among aposematic species: Hypotheses

**Table S1.** Background data on aposematic models from the three butterfly mimicry rings studied

**Figure S2**. Quantification of warning colour patterns.

**Figure S3**. Variation of bird abundance alone the predation pressure gradient

**Table S2**. Comparison of binomial GLMMs fitted to the data on butterfly attack rates

**Table S3.** Effect of background predation pressure (PP) on (**a**) attack rates and (**b**) abundances of butterflies in studied mimicry rings

**Figure S4.** Effect of background predation pressure (PP) on attack rates of mimicry rings

**Table S4.** List of focal species found in the sampling sites belong to three mimicry rings


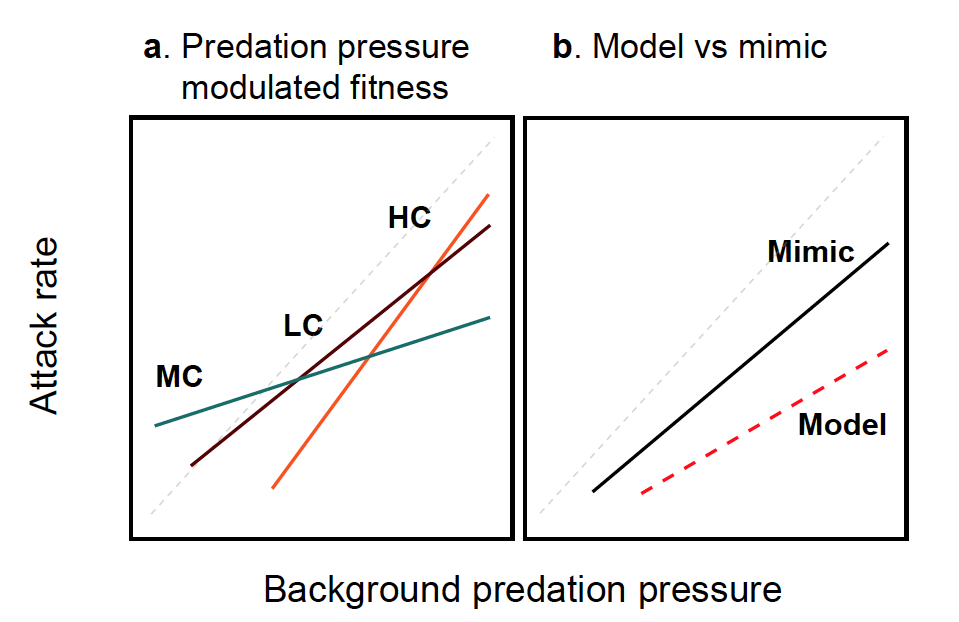


**Figure S1.** Explaining signal diversity among aposematic species: Predicted relationships between background predation pressure and attack rates on focal species according to our hypotheses: **a**. Predation pressure modulated fitness: all species are adequately toxic but signal fitness varies with the background predation pressure. Highly conspicuous butterflies benefit from predator innate avoidance and rapid learning at low background predation pressures, but at high background predation pressures detectability is a disadvantage (especially in the presence of mimics). Conversely, less conspicuous species suffer higher attack rates at low background predation pressure but benefit from crypsis at high background predation pressures. Similar predictions for models and mimics according to Batesian theory: **b**. Attack rates on mimics are always higher than on models, and increase more rapidly with increasing background predation pressure. The grey broken line indicates the attack rate on non-aposematic, non-mimetic species (i.e the background predation pressure). LC – least conspicuous, MC – moderately conspicuous, HC – highly conspicuous.

| **Table S1.** Background data on aposematic models from the three butterfly mimicry rings studied: Pattern simplicity score (PSS) (P = 0.42), conspicuousness (P = 0.00), toxicity (P = 0.64) and wing hardness (P = 0.5) (see Methods section for details). | | | | | |
| --- | --- | --- | --- | --- | --- |
| Ring |  | Mean PSS  (n = 19) | Conspicuousness  (n = 10) | Toxicity (LD50/mg kg^-1^)  (n = 9) | Wing hardness (gf)  (n = 39) |
| *Danaus* |  | 0.179 ± 0.15 | 24.43 ± 0.61 | 134.82 ± 64.58 | 2.31 ± 0.68 |
| *Tirumala* |  | 0.168 ± 0.17 | 21.24 ± 1.96 | 118.42 ± 101.95 | 2.49 ± 0.75 |
| *Euploea* |  | 0.099 ± 0.10 | 19.02 ± 0.05 | 190.83 ± 112.43 | 2.74 ± 1.29 |


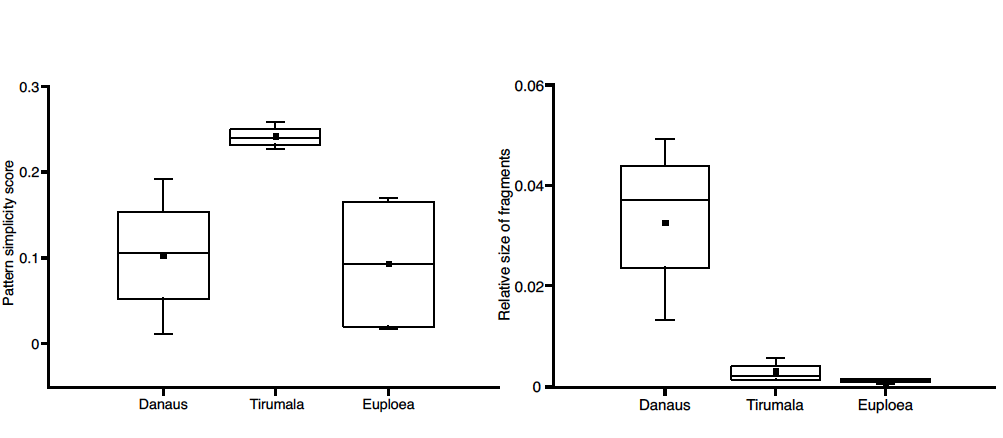


**Figure S2**. Quantification of warning colour patterns. **a**. Pattern simplicity score (PSS - invers of complexity); Lower the values higher the complexity where PSS = 1 is a perfect circle. **b**. Size of warning signal colour fragments (patches) relative to the total wing area. *Danaus* had larger fragments and colour patches on *Tirumala* were smaller while *Euploea* had tiny colour fragments.


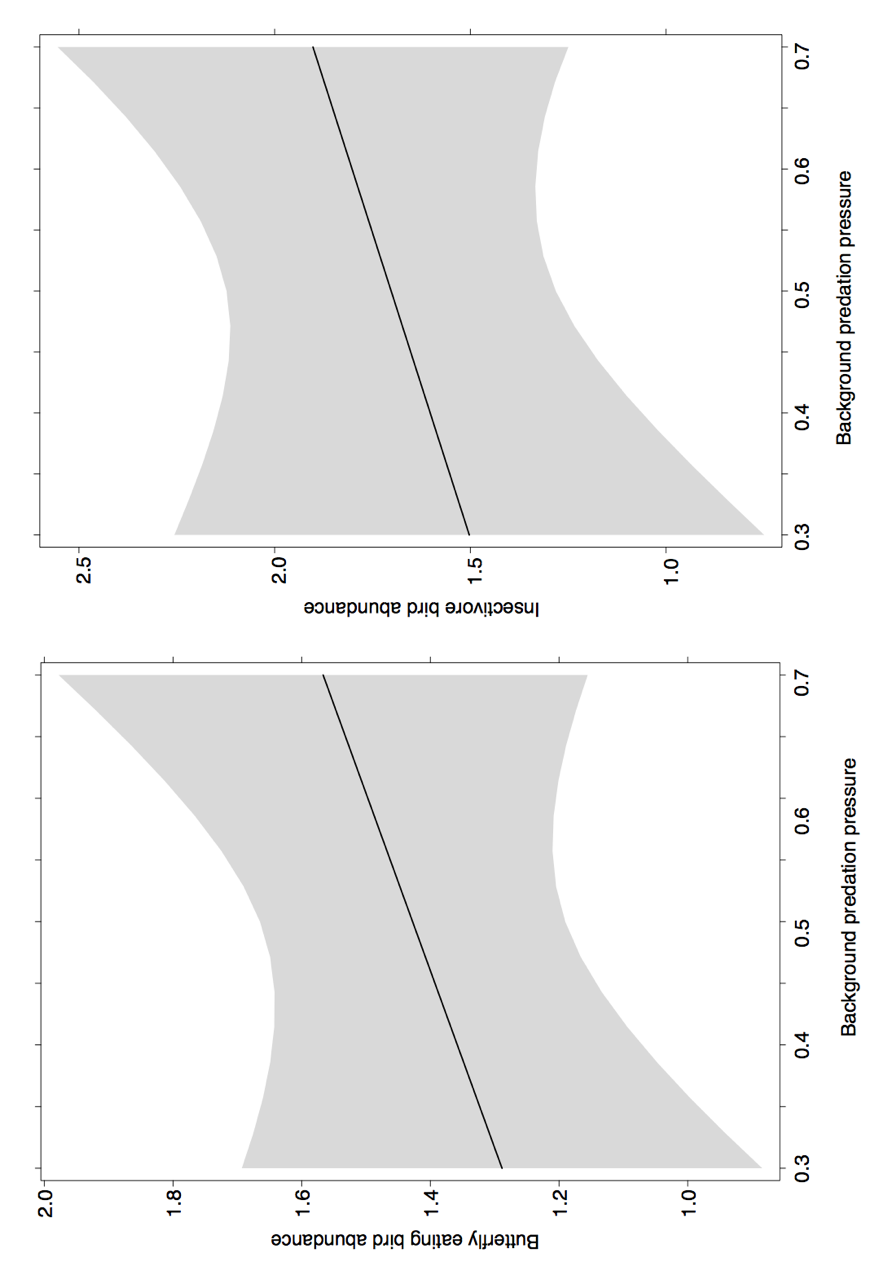


**Figure S3**. Variation of bird abundance alone the predation pressure gradient measured as encounter rates. **a**. Abundance of birds known to eat butterflies b. Abundance of Insectivore birds

| **Table S2**. Comparison of binomial GLMMs fitted to the data on butterfly attack rates (proportion of individuals with bird beak marks) across 11 sites in Sri Lanka and SW China. | | | | | |
| --- | --- | --- | --- | --- | --- |
|  | Model parameters | | AICc | ∆_i_ | W_i_ |
|  | Background predation pressure (PP) + Ring + Model/Mimic + Relative Model/Mimic abundance + PP:Ring + (1\| Site) | | 724.3 | 0 | 0.354 |
|  | PP + Ring + Model/Mimic + PP:Ring + (1\| Site) | | 725.3 | 1.02 | 0.213 |
|  | PP + Ring + Model/Mimic + PP:Ring + Relative edible abundance + (1\| Site) | | 726.4 | 2.10 | 0.124 |
|  | PP + Ring + Model/Mimic + PP:Ring + (1\| Site) | | 726.4 | 2.18 | 0.119 |
|  | PP + Ring + Model/Mimic + PP:Ring + PP : Model/Mimic + (1\| Site) | | 726.4 | 3.07 | 0.076 |
|  | | AICc, Akaike Information Criteria (Corrected). ∆_I,_ Score differences. W_i_ Akaike weights. The full model was: Attack rate ~ Background predation pressure + Relative* edible prey abundance + Relative* model/mimic abundance + Ring + Model/Mimic + Model/Mimic: Background predation pressure + Ring: Background predation pressure + (1\| Site/Transect). * Relative to total prey of all types. | | | |

| **Table S3.** Effect of background predation pressure (PP) on (**a**) attack rates and (**b**) abundances of butterflies in studied mimicry rings presented as the 95% of confidence intervals of fixed effects. | | | |
| --- | --- | --- | --- |
|  | **a**. Formula: Attack rate ~ Background predation pressure (PP) + Ring + ModMim + Relative abundance + relative abundance of edible species + PP:Ring + PP:Model/Mimic + Ring:Model/Mimic + (1 \| Site) | | |
|  | Fixed effects and interaction terms | 2.5% | 97.5% |
|  | (Intercept) | -6.480 | -2.043 |
|  | PP | 5.143 | 13.832 |
|  | Model/Mimic (Model) | -1.177 | 0.051 |
|  | Ring(Tirumala) | 1.224 | 5.918 |
|  | Ring(Euploea) | 0.081 | 4.629 |
|  | PP:Ring(Tirumala) | -12.820 | -3.700 |
|  | PP:Ring(Euploea) | -9.457 | -0.147 |
|  | PP:Model/Mimic | -1.398 | -0.102 |
|  | Relative abundance | -0.560 | 8.988 |
|  | Relative abundance of edible species | -3.292 | 1.193 |
|  | **b.** Formula: Abundance ~ Background predation pressure (PP) + Ring + ModMim + PP:Ring + PP:Model/Mimic + Ring:Model/Mimic + (1 \| Site) | | |
|  | Fixed effects and interaction terms | 2.5% | 97.5% |
|  | (Intercept) | -71.078 | 70.616 |
|  | PP | -107.721 | 147.234 |
|  | Ring(Tirumala) | -105.800 | 62.712 |
|  | Ring(Euploea) | -25.120 | 144.300 |
|  | Model/Mimic (Model) | 0.563 | 141.407 |
|  | PP:Ring(Tirumala) | -114.813 | 178.952 |
|  | PP:Ring(Euploea) | -268.571 | 30.261 |
|  | PP:Model/Mimic(Model) | -227.659 | 13.302 |
|  | Ring(Tirumala): Model/Mimic(Model) | -4.279 | 84.097 |
|  | Ring(Euploea): Model/Mimic(Model) | 6.645 | 94.000 |


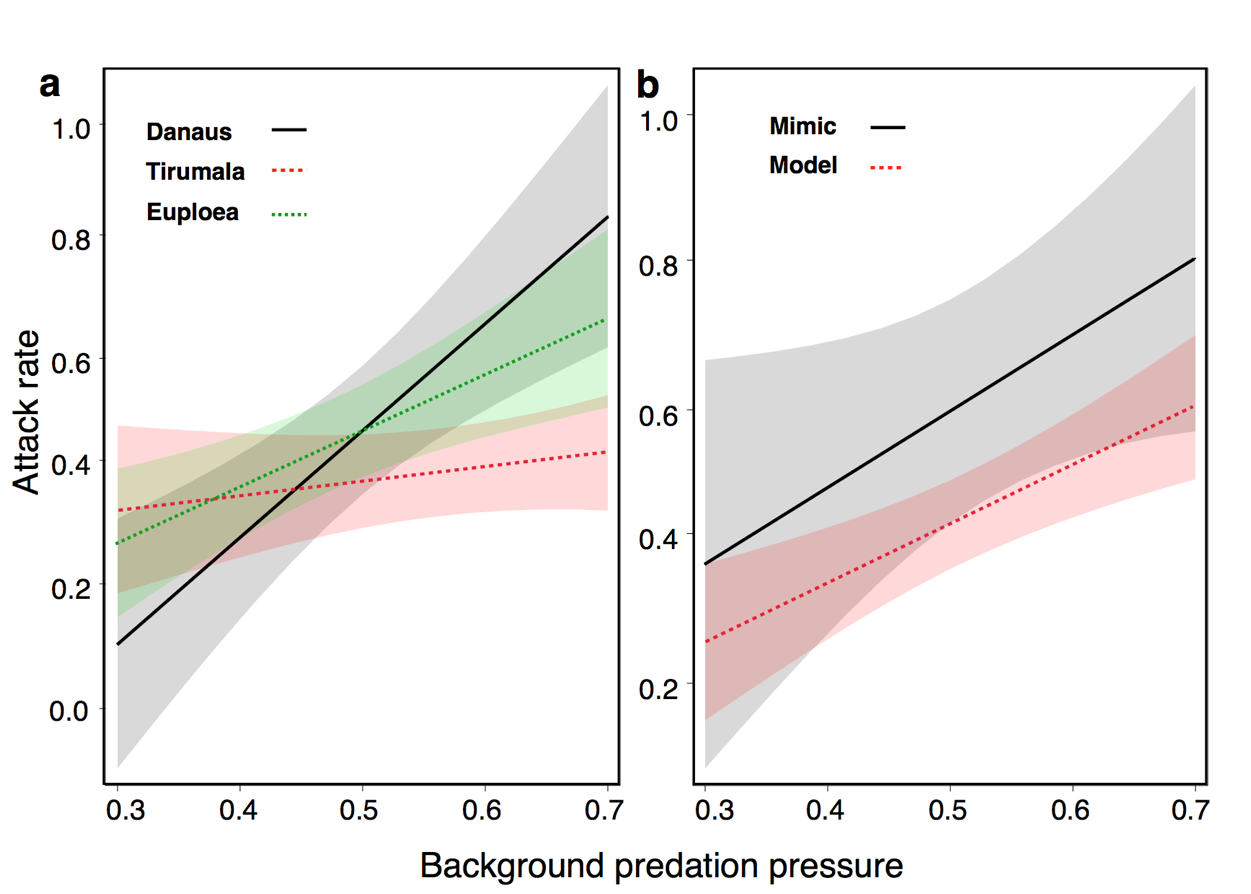


**Figure S4.** Effect of background predation pressure (PP) on attack rates of mimicry rings **a**. Predation pressure modulated fitness: Aposematic (model) butterflies in three mimicry rings undergo different attack rates alone the same PP gradient as predicted **b**. Mimics face higher attack rates than model species and this difference increase with the PP, although this latter effect was not significant. Shaded area shows the 95% confidence intervals.

**Table S4.** List of focal species found in the sampling sites belong to three mimicry rings

| Family/Subfamily | Species | Ring | Model/Mimic |
| --- | --- | --- | --- |
| Nymphalinea | *Argyreus hyperbius* | Danaus | Mimic |
| Nymphalinea | *Euripus* sp1 | Tirumala | Mimic |
| Nymphalinea | *Euripus* sp2 | Tirumala | Mimic |
| Nymphalinea | *Hestina nama* | Tirumala | Mimic |
| Nymphalinea | *Hypolimnas bolina* | Euploea | Mimic |
| Nymphalinea | *Hypolimnas misippus* | Danaus | Mimic |
| Nymphalinea | *Paranticopsis xenocles* | Tirumala | Mimic |
| Papilionidae | *Chilasa agestor* | Tirumala | Mimic |
| Papilionidae | *Chilasa paradoxa* | Euploea | Mimic |
| Papilionidae | *Chylasa clytia* | Tirumala | Mimic |
| Papilionidae | *Chylasa clytia* | Euploea | Mimic |
| Pieridae | *Pareronia ceylanica* | Tirumala | Mimic |
| Satyrinae | *Elymnias hypemnestra fraterna* | Danaus | Mimic |
| Satyrinae | *Elymnias hypemnestra undularis* | Euploea | Mimic |
| Satyrinae | *Elymnias nesaea apelles* | Tirumala | Mimic |
| Satyrinae | *Elymnias Palmfly* | Euploea | Mimic |
| Satyrinae | *Penthema darlisa* | Tirumala | Mimic |
| Danainae | *Danaus chrysippus* | Danaus | Model |
| Danainae | *Danaus genutia* | Danaus | Model |
| Danainae | *Parantica aglea* | Tirumala | Model |
| Danainae | *Parantica melaneus* | Tirumala | Model |
| Danainae | *Parantica sita* | Tirumala | Model |
| Danainae | *Tirumala limniace* | Tirumala | Model |
| Danainae | *Tirumala septentrionis* | Tirumala | Model |
| Danainae | *Euploea core* | Euploea | Model |
| Danainae | *Euploea klugii* | Euploea | Model |
| Danainae | *Euploea midamus* | Euploea | Model |
| Danainae | *Euploea mulciber* | Euploea | Model |
| Danainae | *Euploea sylvester montana* | Euploea | Model |
| Danainae | *Idea iasonia* | Tirumala | Model |
| Nymphalinea | *Cethosia biblis* | Danaus | Model |
| Nymphalinea | *Cethosia cyane* | Tirumala | Model |
| Nymphalinea | *Cethosia cyane* | Danaus | Model |
| Nymphalinea | *Cethosia nietneri* | Danaus | Model |
